# Resilient infrastructure

**DOI:** 10.1038/s44172-022-00029-0

**Published:** 2022-11-03

**Keywords:** Civil engineering

## Abstract

Today we present a special issue of *Communications Engineering* exploring various ways in which engineering researchers are engaging with the challenge of building resilient infrastructure for a sustainable future.

The entire world is undertaking huge infrastructure projects. There are high speed rail lines being built in the UK^1^, China^2^, and Mexico^3^. The Biden-Harris administration has pledged $550 billion of investment into infrastructure to rebuild roads and bridges, modernize ports and airports, replace lead pipes to deliver clean water, and expand high-speed internet^4^. In Germany, sustainable infrastructure is changing the urban fabric of the capital, Berlin, in an 8 billion Euro redevelopment project^5^. These projects are immense political, technological, and economic drivers which will undoubtedly impact the health, mobility, connectivity, and comfort of billions of people worldwide. But how should we build such infrastructure?

Some say that things just aren’t made how they used to be. But sometimes the way we make things needs to change. Resilience is the capacity to withstand, prepare for, recover from and adapt following disruption. It is a central tenet of the Sustainable Development Goals, from safe settlements and built structures to the secure supply of energy, water and food. Thus it is a major challenge for engineers who need to re-think how we design and build infrastructure to achieve the sustainable world we want.

This special issue draws together the insights of researchers from different fields who think about resilient infrastructure in different ways. In a Viewpoint, four of our editorial board members offer their perspective on key challenges and research needs to achieve resilient water supply, built environment, communications networks and supply chains. In a Comment piece, our editorial board member Dr. Danielle Densley Tingley discusses the importance of circular economy thinking to create long lasting housing stock. In a Q&A with Professor John Provis, we explore the future of cement technology, which touches on issues of durability for longevity and the use of local raw materials for more resilient supply chains. We also present two Research Highlights on papers published in the *Nature* Portfolio. One describes how extending power grids to increase capacity might counterintuitively place additional pressure on different parts of the network. The other Research Highlight looks at the importance of mapping local subsidence in coastal regions to identify a clearer picture of relative sea level rise, a critical issue to consider when planning urban development. Finally, a research paper from Thomas Matarazzo and colleagues published today in *Communications Engineering* explores the potential to use crowdsourced smartphone data to monitor the modal frequencies of bridges, as a route to cheap and continuous structural health monitoring of these vital components of transport infrastructure.

Some key challenges resonate across different parts of the Special Issue. One issue is the vulnerability of the poorest communities to weak infrastructure in the face of disaster. For example, during the Covid pandemic, breakdowns in supply chains meant that food and medicines were harder to source and access and more expensive to buy. And limitations in network connectivity in poorer communities meant children could not access online education. During natural disasters large or small, it is the poorer communities who suffer the greatest livelihood disruption. Also emergent is the importance of a systems approach to resilience considering the inter-dependence of different infrastructure systems. To take a simple example, a single building resisting an earthquake is not resilient if all the utilities supporting life within the building are destroyed. and the surrounding environment turned to rubble. A third strand is the importance of predicting the impact of resilience measures in dynamic systems to enable implementation of the most effective measures.

Emerging engineering opportunities for enhancing resilience are coming from exploiting data from communications networks. These data can be used to identify alternative supply chains or to monitor structural health. However, increasing the resilience of our communications networks to cyberthreats is also an important need. Technology integration is also an exciting opportunity, for example water treatment technologies are being developed which harness and even generate a sustainable energy supply.

One of our key goals for *Communications Engineering* is to leverage the journal’s multidisciplinary scope to bring together researchers working in different ways towards similar goals^6^, to create a sense of a shared mission, to look for synergies, and to create opportunities. With this content we have taken a first step in this direction. The articles will be gathered in a special collection on our Collections webpage and we will continue to add content as we publish more papers on this topic. We invite you to submit to *Communications Engineering* your engineering research into approaches to make infrastructure stronger, safer and more sustainable.
